# Difficulty in Assigning Fungal Identity Based on DNA Sequences

**DOI:** 10.1128/MRA.00460-21

**Published:** 2021-12-02

**Authors:** Oscar N. Ruiz, Osman Radwan

**Affiliations:** a Fuels and Energy Branch, Aerospace Systems Directorate, Air Force Research Laboratory, Wright-Patterson AFB, Ohio, USA; b Environmental Microbiology Group, University of Dayton Research Institute, Dayton, Ohio, USA; Vanderbilt University

## REPLY

The Comment Letter by Houbraken et al. (1) correctly points out the need for accurate fungal taxonomical identification in biological research and the difficulty confronted by scientists in assigning genus and species identity due to the high genome sequence similarity of related individuals and the low availability of full fungal genome sequences in DNA data repositories with which to perform sequence comparisons. The Comment Letter accurately presents the need to revise or even update previously assigned fungal classifications as more DNA sequences and full genomes become available over time. The Comment Letter highlights that currently, the internal transcribed spacer (ITS) region is most often used to identify fungi, and the authors argue that some short regions of genes such as *benA*, *caM*, and *tef*1-alpha may be better suited for accurate species identification of close relatives. Based on that premise, the authors used the partial beta-tubulin (*benA*) and partial calmodulin (*caM*) gene sequences to reassess the taxonomical identity of multiple fungal isolates published in journals such as MRA (previously known as *Genome Announcements*) using phylogenetic trees. The authors argue that upon analysis of the short regions of the *benA* or *caM* genes, 21 published papers in MRA had incorrectly assigned the fungal identity of their isolates. While we agree that assigned fungal taxa should be revised as more DNA sequence information becomes available, this does not mean that the classification provided at the time of publication was erroneous based on the available sequence information at the time. Furthermore, we argue that the authors actually contradicted their own argument when they ignored available ITS sequences, 18S rRNA gene sequences, and even full genome sequences and relied on a very short 65-amino acid region of the *benA* gene to assign fungal identity. Based on the 65-amino acid region of *benA*, Houbraken et al. (1) claimed that our paper (2) misidentified the isolate as *Byssochlamys* sp., when it should have been Monascus floridanus.

We argue that the analysis performed by Houbraken et al. (1) should have taken into consideration the ITS, full 18S rRNA gene, and whenever possible, the alignment of any genome sequences available to provide the most accurate classification possible and not rely on a small portion of the *benA* gene. While we understand that taxonomical identification may improve as more DNA sequence data become available, potentially leading to a change in a fungal isolate identity, we strongly believe that the *Byssochlamys* sp. classification provided by Radwan et al. (2) for isolate BYSS01 (GenBank accession number NIXA00000000) was accurate at the time and stands correct today until further characterization is provided. Still today, isolate BYSS01 has Byssochlamys fulva (AB023941.1) as the closest hit from a BLAST alignment of the full-length (2,105-bp) 18S rRNA gene, with 94.2% sequence similarity in 100% of the query sequence and a maximum score (highest alignment score) of 3,197. However, the closest species of *Monascus* was M. fuliginosus (HM188432.1), which came in at 29th place in the BLAST analysis with a maximum score of 2,034 and only presenting sequence similarity in 82% of the BYSS01 query sequence. In fact, the full-length 18S rRNA gene of *M. fuliginosus*, M. purpureus (HM188427.1), and M. ruber (HM188429.1) is only 1,751 bp long with a 354-bp deletion between positions 602 and 978 of the BYSS01 18S rRNA gene sequence. Furthermore, the available *benA* sequence (KY709172.1) of *M. fuliginosus* only covers 8% of the total BYSS01 BenA protein, precluding a true comparison. For this reason, we argue that the change of genus and assignment of species classification by Houbraken et al. (1) is premature, based on the limited information presented in the Comment Letter.
